# Experiences of Family Caregivers Providing Home Care to Older Patients With Cancer: A Qualitative Study

**DOI:** 10.1097/jnr.0000000000000579

**Published:** 2023-11-24

**Authors:** Cheng-Fang YANG, Chien-Ning TSENG, Yuan-Ju LIAO, Zi-Xuan GAO, Hsiao-Ping CHEN, Po-Chih CHANG, Yun-Hsiang LEE

**Affiliations:** 1 PhD, RN, Assistant Professor, Second Degree Bachelor of Science, College of Medicine, National Taiwan University, Taipei, Taiwan; and Adjunct Supervisor, Department of Nursing, National Taiwan University Hospital, Yunlin Branch, Yunlin, Taiwan; 2 PhD, RN, Assistant Professor, Department of Nursing, Asia Eastern University of Science and Technology, New Taipei, Taiwan; 3 RN, Doctoral Candidate, Department of Nursing, National Yang Ming Chiao Tung University, Taiwan; 4 MSN, RN, Department of Nursing, National Taiwan University Hospital, Taipei, Taiwan; 5 MSN, RN, Head Nurse, Department of Nursing, College of Medicine, National Taiwan University Hospital, Taipei, Taiwan; 6 BSN, RN, Department of Nursing, College of Medicine, National Taiwan University Hospital, Taipei, Taiwan; 7 PhD, RN, Assistant Professor, School of Nursing, College of Medicine, National Taiwan University, Taipei, Taiwan; and Adjunct Head Nurse, Department of Nursing, College of Medicine, National Taiwan University Hospital, Taipei, Taiwan.

**Keywords:** cancer, family caregiver, older patient, older adults, at-home care

## Abstract

**Background:**

Older patients with cancer receive anticancer therapy in outpatient settings, and care-related issues may occur after discharge, which often requires family caregivers (FCs) to play a significant role in providing cancer care at home. However, relatively few studies have been focused on exploring the care experiences of these FCs.

**Purpose:**

The aim of this study was to explore the care experiences of FCs caring for older family members with cancer at home.

**Methods:**

A qualitative study design and in-depth individual interviews were used to explore the at-home care experiences of FCs of older patients with cancer. The research was conducted in chemotherapy outpatient settings of a medical center in northern Taiwan. Content analysis was used to analyze data. The analyses focused on first extracting meaningful units from the text and then inducting categories from these units and determining the major themes.

**Results:**

Twenty FCs were interviewed. The three themes identified included (a) increased information needs and challenges in diet preparation and treatment decision making, (b) personal and patient-induced emotional stress, and (c) life rebalancing through the care experience.

**Conclusions/Implications for Practice:**

The findings highlight the educational requirements, especially related to meeting personal dietary needs and obtaining psychological support, for FCs caring for older patients with cancer to help them rebalance their life.

## Introduction

Most developed countries define older people as individuals aged 65 years or over (World Health Organization, 2018). On the basis of this threshold, older adults account for 16.85% of Taiwan's total population, meeting the widely accepted definition of an “aged society” (Ministry of the Interior, Taiwan, ROC, 2022). In an aging society, the incidence of cancer, diabetes, neurodegenerative and cardiovascular diseases, and other serious ailments is higher in aging societies, with cancer ranking as the largest cause of morbidity in older adults and the mean age of death from cancer being 70 years. Most older patients experience comorbid chronic diseases (Ministry of Health and Welfare, Taiwan, ROC, 2021). In addition, because of age-related declines in physical functioning (Akechi et al., 2012; Derks et al., 2016; Nightingale et al., 2021), hospitalization-related costs, durations, comorbidities, and mortality tend to be higher in this group as well (Gbeasor-Komlanvi et al., 2020). Moreover, probability of rehospitalization increases with patient age and cancer stage (Stitzenberg et al., 2015). In light on the above factors, caring for older patients with cancer is highly challenging for the healthcare system and for caregivers. Furthermore, the results of prior research indicate that caregivers of older cancer survivors experience higher levels of distress, a lower quality of life, and greater anxiety than the general population (Jansen et al., 2018).

As responsibility for managing cancer-related symptoms increasingly shifts from hospital to family settings, family caregivers (FCs) must necessarily take on care responsibilities originally assigned to professional staff during hospitalization, including managing pain, nausea, fatigue, and medications and deciding whether and when to contact physicians (van Ryn et al., 2011). A systematic review on older patients with cancer found the highest unmet social support need to be medical support (39%), followed by informational (35%), physical (30%), emotional (28%), and practical (20%) support needs (Kadambi et al., 2020). The priority healthcare education needs of FCs of older patients with cancer (Bangerter et al., 2019; Gröpper et al., 2016) focus on drug and nutrition information, home self-care, and assisting patients adjust their comfort (Ghorbani et al., 2020; Hestevik et al., 2020). Thus, after older patients with cancer are discharged home from the hospital, it is vital for FCs to have sufficient information, caregiving skills, and support.

Currently, many older patients with cancer return home to rest after outpatient treatment and to wait for the next cycle of anticancer therapy, where they are cared for by family members. This results in immense stress for FCs, as only professional staff can comprehensively evaluate the healthcare needs of these patients (Baider & Surbone, 2014). FCs are nonformal caregivers (e.g., spouses, family members, friends, or neighbors) who generally care for the health and daily lives of patients without remuneration. In addition, many FCs are required to provide physical and psychological support as well as financial assistance (S. H. Kim et al., 2018). These various care tasks cause immense psychological stress, resulting in anxiety and depression among FCs (Lee et al., 2013). Studies have found that family members caring for older patients with cancer exhibit more anxiety and depression, lower levels of perceived social support, and poorer coping capacity than caregivers of older patients without cancer (Goldzweig et al., 2013). Moreover, duration of care increases the perceived burden of FCs. Patients' lack of appetite and depressive emotions, as well as caregivers' fatigue, pain, and lack of confidence in their caregiving work, increases the burden on caregivers (Lee et al., 2018). Therefore, 25% of family members adjust their work hours, take leave, or quit their jobs to care for patients (Wadhwa et al., 2013).

Relatively few studies have focused on exploring the experiences of FCs in caring for their older family members with cancer at home. In this study, qualitative methods were employed to investigate these experiences to elucidate a more in-depth understanding of the actual contextual experiences of these caregivers, which is important for developing and implementing concrete support strategies for FCs.

## Methods

### Research Design

The topic was approached in this study from a qualitative perspective. To understand participant experiences, it is necessary to get acquainted with their world and explore the nature of phenomena (Holloway & Galvin, 2016; Speziale et al., 2011). Qualitative research allows researchers to increase understanding of human experiences, which is crucial for health professionals who focus on caring, communication, and interaction (Holloway & Galvin, 2016; Speziale et al., 2011). Thus, we studied the experiences of FCs in caring for patients with cancer. A qualitative study design was used to explore the at-home caregiving experiences of 20 FCs of older patients with cancer. In-depth interviews were conducted to provide participants with the opportunity to fully describe their experiences.

### Sample

The inclusion criteria were FCs who (a) were over 18 years old, (b) were caring for older cancer patients receiving anticancer therapy in a chemotherapy outpatient setting, (c) were identified by their patient as their primary caregiver, and (d) lived in the same house with their patient.

### Ethical Considerations

The institutional review board of National Taiwan University Hospital in Taipei approved this study (number: 201809058RIND). The purpose of the study was explained to all of the participants, and they were all informed of their rights, including that they could withdraw from the study at any time. All of the participants signed an informed consent form.

### Data Collection

FCs of older patients with cancer were approached from January to December 2019 in the chemotherapy outpatient setting of a medical center in northern Taiwan and invited to participate in this study. A trained researcher (interviewer) with 4 years of oncology nursing experience interviewed 20 patients with cancer. The FCs were informed that they were being digitally recorded during the interview and received FC consent to do so. The interviewer used face-to-face, open-ended questions to elicit responses. Examples of these questions include “Could you share with me your experiences of caring for older cancer patients at home?”, “Feel free to state your thoughts or opinions on [topic],” and “What impressed you most during your caregiving experience?” If a participant described hardship in caring for a patient, they were asked, “How did you adjust the situation by yourself?” To ensure the participants were more likely to express their authentic feelings, the interview was conducted in a quiet, private clinic room during chemotherapy or after consulting with a doctor.

Twenty FCs were enrolled as participants (see Table 1). The duration of each interview was approximately 30–90 minutes. Data collection reached saturation at the 20th interview, when the relationships among the meaning units, categories, and themes were similar in the newly collected data and no new information was extracted from the interview.

**Table 1.** Patient Characteristics (*N* = 20)

ID	Age (Years)	Gender	Education	Marital Status	Diagnosis	Stage	Current Treatment				Chronic Disease
							Chemo	Radio	Targeted	Immuno	
1	77	Male	Elementary	Married	Gastric	IV	Yes	No	No	No	H/T
2	68	Male	Bachelor	Married	Esophageal	III	Yes	Yes	No	Yes	H/T, DM, HD
3	74	Male	High school	Married	Lung	IV	Yes	No	Yes	Yes	
4	75	Male	High school	Married	Bladder	II	Yes	Yes	No	No	H/T, HD
5	70	Male	Elementary	Married	Esophageal	III	Yes	Yes	No	No	HD
6	68	Female	None	Married	Colorectal	IV	Yes	No	Yes	No	
7	82	Male	Bachelor	Married	Colorectal	III	Yes	No	Yes	No	DM, HD
8	80	Male	Elementary	Married	Colorectal	III	Yes	No	Yes	No	
9	70	Male	Associate	Married	Lung	II	Yes	No	No	No	
10	72	Male	High school	Married	Colorectal	IV	Yes	No	Yes	No	Parkinson
11	69	Female	None	Married	Lung	III	Yes	No	Yes	No	DM
12	84	Male	Elementary	Married	Lung	III	Yes	Yes	Yes	No	H/T, HD
13	77	Female	Elementary	Married	Lung	IV	Yes	No	Yes	No	H/T, DM
14	75	Female	Elementary	Married	Colorectal	III	Yes	No	No	No	H/T, HD
15	96	Female	Elementary	Widowed	Lymphoma	III	Yes	No	Yes	No	
16	66	Male	Bachelor	Married	Cholangiocarcinoma	IV	Yes	No	No	No	Hepatitis C, liver cirrhosis
17	76	Female	Elementary	Married	Colorectal	IV	Yes	Yes	Yes	No	
18	79	Female	Elementary	Widowed	Schwannoma	IV	Yes	No	Yes	No	H/T
19	71	Female	Elementary	Married	Lung	III	Yes	No	No	Yes	HD
20	76	Female	Elementary	Married	Gastric	IV	Yes	No	No	No	H/T

*Note.* Chemo = chemotherapy; Radio = radiotherapy; Targeted = targeted therapy; Immuno = immunotherapy; H/T = hypertension; DM = diabetes mellitus; HD = heart disease.

### Data Analysis

Data analysis was conducted using content analysis (Graneheim & Lundman, 2004). The participants, who were of diverse genders and ages, expressed their caregiving experiences. The findings were confirmed by the authors, who were all trained in qualitative research, through peer debriefing. The interviews were audio-recorded, and detailed field notes were taken. The text was reviewed word-by-word.

The analysis steps form a well-known method that determines the presence of codes, categories, and themes within text data in a qualitative study (Graneheim & Lundman, 2004). Two of the authors read and analyzed all of the interviews independently. These two researchers read all of the transcripts repeatedly to immerse themselves in the caregiving experiences of the participants to achieve an authentic sense of these experiences. The analyses focused on extracting meaningful units from the text and then inducting categories from these units. The authors discussed and reached an agreement on how to label the themes.

### Rigor

Data trustworthiness was ensured using several strategies (Graneheim & Lundman, 2004; Lincoln & Guba, 1985). For credibility, the first author was trained to conduct qualitative interviews and used face-to-face, open-ended questioning. Peer debriefing was used to ensure that themes and categories were substantiated by the data. An audit trail of all documents used in data collection and analysis was recorded and made accessible to ensure dependability. Rich descriptions and the data analysis procedure were described in detail to ensure confirmability. Detailed descriptions of the research situation and methods were provided to help assess the transferability of the findings and meanings to others in similar situations.

## Results

### Demographics

Twenty FCs of older patients with cancer at home were enrolled as participants and interviewed in this study. Their demographics are presented in Tables 1 and 2. In terms of patients, the average age was 75.3 years; nine were women, and 11 were men; and over half had either colorectal (30%) or lung (30%) cancer. All of the patients were receiving chemotherapy, with some also receiving other treatments such as radiotherapy, targeted therapy, and immunotherapy. Most (70%, *n* = 14) also had chronic diseases (Table 1).

**Table 2.** Family Caregiver Characteristics (*N* = 20)

ID	Age	Gender	Education	Marital Status	Relationship With Patient	Occupation	Previous Experience	Daily Care Time (Hours)	Duration (Months)	Care Model
1	46	F	Bachelor	Married	Child	Part-time	No	2	6	Nonindependent care all day
2	64	F	High school	Married	Spouse	Unemployed	No	18	7	Alternative care
3	65	F	High school	Married	Spouse	Unemployed	Yes	24	7	Independent care all day
4	40	M	Bachelor	Single	Child	Unemployed	No	3	6	Nonindependent care all day
5	39	F	Bachelor	Married	Child	Part-time	No	6.5	2	Alternative care
6	47	F	High school	Divorced	Child	Employed	Yes	7	6	Alternative care
7	77	F	Bachelor	Married	Spouse	Unemployed	No	24	48	Independent care all day
8	48	F	High school	Married	Daughter-in-law	Employed	No	7	12	Alternative care
9	70	F	Associate	Married	Spouse	Employed	No	8	1	Independent care all day
10	63	F	High school	Married	Spouse	Unemployed	Yes	24	132	Independent care all day
11	42	F	Associate	Married	Child	Employed	No	7	60	Alternative care
12	40	F	Bachelor	Single	Child	Employed	No	1	12	Alternative care
13	54	F	High school	Married	Child	Unemployed	No	3	36	Alternative care
14	52	F	Associate	Married	Daughter-in-law	Unemployed	No	4.5	6	Nonindependent care all day
15	65	F	Doctor	Married	Child	Employed	Yes	5	6	Nonindependent care all day
16	56	F	Bachelor	Married	Spouse	Unemployed	No	20	6	Independent care all day
17	46	F	High school	Divorced	Child	Unemployed	No	24	8	Independent care all day
18	49	F	Doctor	Married	Child	Part-time	No	24	12	Independent care all day
19	46	M	Bachelor	Married	Child	Employed	No	8	2	Alternative care
20	26	F	Master	Single	Grandson	Unemployed	No	24	2	Nonindependent care all day

*Note.* F = Female; M = male.

In terms of the participants (FCs), their average age was 51.8 years, most were female (90%, *n* = 18) and were either the child (55%, *n* = 11) or spouse (30%) of the care recipient, three quarters (75%, *n* = 15) were married, and half (50%, *n* = 10) were employed either full- or part-time. Most of the participants did not have previous caregiving experience (80%, *n* = 16). The average number of hours of care provided per day was 12.2 (*SD* = 9.1), and the average duration of care was 18.9 months. One third (35%) of the participants did not have another family member who could also help with caregiving and were thus responsible for providing full-day independent care (Table 2). The three themes that emerged from the content analysis results are summarized in Table 3.

**Table 3.** Caregiving Experiences of Family Caregivers of Older Patients With Cancer at Home

Theme	Category	Example of Quotes
1. Increased information needs and challenges in diet preparation and treatment decision making	(1) Diet preparation as a major challenge	*It is more difficult for me to prepare food for the patient….* (C11) *We have been challenged with all kinds of cooking methods.... He (patient) drank soup because drinking soup is easier for him....* (C14)
	(2) Lack of information and medical knowledge for decision making	*He did not say anything [about the treatment plan.] “How could we make decisions then?” He should [have] provide[d] some information so that we could make correct judgments….* (C15)
2. Personal and patient-induced emotional stress	(1) Patient effect on family caregiver emotions	*Sometimes, I also feel unwell, and he would get mad and shout at me [from upstairs]. But [However,] he would soon come down….* (C7)
	(2) Family caregivers lose patience	*Sometimes I would lose my cool [become angry] and patience to an extent…. However, I knew that she was sick and feeling unwell. “What could I do?”…* (C17)
3. Life rebalancing through the care experience	(1) Finding methods for relieving stress	*I am a cheerful person, and I listen to music and watch TV dramas…. Those activities are easy methods [ways] for me to release [my stress]….* (C10)
	(2) Changes in the modes of interaction	*I took care of him [the patient] like a baby. I treated him as if he was a glass doll…an infant, [as though he was] a mother who just gave birth and sitting the month.* (C16)
	(3) Adjusting to a new pace of life	*There is no choice, you must adjust it, so I have re-arranged all my schedules.* (C3)

*Note.* FC = family caregivers.

### Increased Information Needs and Challenge in Diet Preparation and Treatment Decision Making

Despite the easy access to information in modern society, medical knowledge tends to be relatively complex and professional in nature. Thirteen (65%) of the participants identified diet preparation as a major challenge in their care experience, and eight (40%) noted they lacked accurate medical knowledge for decision making. Medical professionals should endeavor to provide clearly understandable information to patients with cancer and their families to enable them to make appropriate treatment decisions, which may increase treatment effectiveness.

#### Diet preparation as a major challenge

Cancer treatment is a long and painful process. A patient's body is weakened by cancer, and they must cope with the side effects of medical treatment. Patients with cancer often experience side effects such as an altered appetite, lack of appetite, and nausea. A major challenge for caregivers is motivating patients with cancer to eat to improve their nutritional status and enhance their immune system. Sixty-five percent of the participants indicated that older survivors of cancer require dietary knowledge and suggested hospitals should provide more information on nutrition.

A 54-year-old participant who had cared for a patient with lung cancer (77 years old) for 3 years stated:

*What our family does not know is what kind of food should be prepared. She has lung cancer. “What are the foods that should be prepared for her?” “Which foods are better for her?” We felt inadequate about this aspect of caregiving. Can the hospital provide more healthy recipes or…?* (C13)

A participant who had cared for her mother for 2 years and had been diagnosed with schwannomas in her left hand for 1 year stated that her mother was a fussy eater, which increased the difficulty of providing care. She stated:

*The difficult aspect is…hmmm…[her] diet. It was extremely difficult…. She was a very picky eater. I could hardly find anything for her to eat. That is right. She usually cooked for herself…. I could only try to convince her to eat high-protein food.* (C18)

Patient tastes may change because of their disease. To encourage their patients to eat more and stay healthy, caregivers must innovate and continually make changes to their patient's diet or attempt new cooking methods. One participant who had provided care to her mother-in-law with colorectal cancer for 6 months indicated that preparing food for her patient was difficult as the patient was physically weak and had poor dental conditions. She stated:

*She (the patient) felt that…her teeth and gum[s] were not connected. She felt that chemotherapy was very tiring. There are things that are necessary for her to do…but she couldn't do them. She could hardly chew and barely ate…. We experimented with various cooking techniques…. She mostly only ate soup because it was easier for her….* (C14)

A 77-year-old participant stated that her husband (82 years old) had colorectal cancer for 2 years. After he became ill, his appetite declined, and she felt stressed by having to prepare three meals a day. She could not sufficiently vary his diet. She stated:

*(The patient's) appetite was poor. No one could blame him…. I could not think of what to prepare. I did not know that I would run out of ideas. Now, I do not know what to do.* (C7)

#### Lack of information and medical knowledge for decision making

Favorable doctor–patient interactions and communications may benefit patient treatments. Family members' emotions should also be considered because they find it difficult to make decisions when affected by doubts. Many (40%) of the participants indicated they lacked the accurate medical information necessary to make decisions. A 65-year-old daughter who had provided care for 6 months to her mother with lymphoma expressed feeling emotionally affected and panicked when her mother was first diagnosed. However, she did not receive further explanations from her doctor and felt hurt. She stated:

*Uh…Initially, when we first received the diagnosis, we felt a mix of emotions. In addition, we did not understand the disease, so we had many questions. At the beginning, [the doctor] did not address our problems, give us direction, or provide us with good suggestions. He did not say anything [about the treatment plan.] “How could we make decisions then?” He should [have] provide[d] some information so that we could make correct decisions. I feel that the counseling aspect was implemented very poorly…. I know that the doctor did not like my communication attitude....* (C15)

Another participant stated that her 70-year-old father had esophageal cancer for 2 months. As the cancer had only been recently diagnosed, there were many treatments that family members needed to understand, discuss, and decide upon. They did not know what the best decision was because of incomplete information. She stated:

*The current situation in choosing a treatment is first understanding the possible impacts of each treatment…A, B, and C. The side effects should be explained before making decisions. It should not, well, I definitely...I don't want radiotherapy. Afterward, the radiotherapy doctor told us about (the side effects)….* (C5)

### Personal and Patient-Induced Emotional Stress

Coping with disease is a stressful situation that affects not only patients but also their caregivers, who experience emotional situations and physiological and psychological stress. Not all of the participants reported experiencing emotional fluctuations while caring for patients, with 45% reporting emotional pressure from patient care and being affected by their patient's emotions.

#### Patient effect on family caregiver emotions

The tedious nature of the cancer treatment process is a challenge for both FCs and patients, requiring both to have a strong will and patience. Some of the participants in this study reported experiencing emotional instability while caring for their patient. A 64-year-old woman had been providing care to her husband with esophageal cancer for 7 months expressed that, during the caregiving process, her greatest source of stress originated from her fear that her husband could die suddenly. Living with this level of uncertainty and anxiety was a miserable experience for her. She did not dare inform her mother-in-law (who was residing in the United States and was over 90 years old) regarding her husband's condition. She stated:

*I would panic suddenly. I thought about him and his disease…. I was always nervous and anxious. I wondered if he might suddenly die in his sleep…. Also, my mother-in-law did not know about this. We did not let her know (chokes).* (C2)

A 46-year-old participant who had taken care of her father with stomach cancer with her mother for 6 months said she could not communicate with her father since his cancer diagnosis. She sometimes argued with him because he had a bad temper and communication was poor after falling sick. She stated:

*He was not so bad-tempered before... (After getting sick) he became very stubborn.... His emotions are up and down.... For example, we told him may not take the medicine, but he insisted on taking it.... I argued with him when I encountered something like that….* (C1)

Sometimes the emotional pressure came not from caring for the patient but because of problems among FCs. A 54-year-old female caregiver said disputes often occurred with her siblings related to caring for their mother. She stated:

*For this (caring for the patient), my brother and sister quarreled.... Taking care of the elderly is a big problem. For example, we all have our own jobs....* (C13)

#### Family caregivers lose patience

A participant who had provided care to her mother with colorectal cancer for 8 months said she often lost her patience. However, reminding herself that she was caring for a patient helped give her the strength to continue. She stated:

*Sometimes I would lose my cool [become angry] and patience to an extent…. However, I knew that she was sick and feeling unwell. “What could I do?” Right…I simply tolerated her and did not take things to heart…. I asked her if she was trying my patience.* (C17)

A 77-year-old female caregiver whose children all resided abroad had been providing care to her husband with colorectal cancer. It was a burden for her. She had been caring for him for 4 years, and she felt unable to have any respite or share her responsibility with anyone. She stated:

*Right now, I am tired. Sometimes, I also feel unwell, and he would get mad and shout at me [from upstairs]. But he would soon calm down. He was already quick-tempered by nature, and I could do nothing about it. Taking care of him tires me out. I have no time to rest…. Sometimes he would get up three or four times at night, which tired me out. I would wonder if there was anyone who could help, but there was no one I could turn to.* (C7)

### Life Rebalancing Through the Care Experience

Families of patients with cancer may alter their lifestyle and methods of interaction in response to conditions during treatment. The most crucial challenge for the caregivers' families was how to adjust and establish an operation model for balancing their family life with disease treatment. Most (85%) of the participants expressed that they had sought ways to release stress, change their interaction modes, and adjust the pace of life while continuing to care for their patient.

#### Finding methods for relieving stress

Providing care to a patient for extended periods may overburden caregivers. By identifying stress-relieving methods, caregivers may manage their emotions and ability to continue performing caregiving tasks. Seven of the participants reported seeking some way to relax, for example, watching TV, swimming, and pursuing religion, during their process of care. A patient with colorectal cancer comorbid with metastatic liver cancer for over a year was cared for by his wife and a foreign helper. As the patient also had Parkinson's disease, he developed hallucinations and became paranoid. His wife was under immense pressure and relieved her stress by listening to music, watching TV, and even shouting. She stated:

*I am a cheerful person, and I…I listen to music and watch TV dramas…. Those activities are easy methods for me to release [my stress]. When my daughter wasn't at home, I would go to her bathroom, which was at the back of our home, and I would shout into the toilet…to release (my bad mood)….* (C10)

A 54-year-old female caregiver had taken care of her mother with lung cancer for 3 years. During that period, she often went to a temple to pray and seek blessings from the gods. She felt that religion provided a source of stability. She stated:

*The power of religion was really helpful. Really…I prayed that the Buddha will let her live a few more years…. I also felt calmer.* (C13)

A participant who had provided care to her husband with colorectal cancer for 4 years together with her son remarked that, because of the tedious nature of the caring process, she sought to relieve her stress through various activities. She stated:

*When I had time, perhaps an hour or two, I would swim [to] relax.* (C7)

#### Changes in the modes of interaction

Caregivers play a crucial role in their patients' treatment process, and their emotions are influenced by the characteristics of their patients. As caregivers interact with patients for extended durations, they must manage their emotions or even change their mode of interaction to facilitate the treatment process. Some of the participants said they needed to be careful during the interactive process and, for example, took care of their patient “like a baby” by using coaxing words. A 40-year-old female caregiver who had cared for her father with lung cancer for over a year stated:

*The hardest part was having to coax him to do things or having to lie to him. My younger sister first attempted to coax him to do something. After she failed, we threatened him. Finally, the three of us took turns to threaten and coax him to do what we wanted him to do. One played the good cop, and the other played the bad cop. Something like that. Through coaxing and lying, we got him to continue his treatment. After he received treatment for approximately three months, he was finally able to accept that he had cancer.* (C12)

One participant shared that her father had multiple cancers and a bad temper and that his interactions with family members often ended in conflict. In response, the family changed its strategy to convince him to accept his situation. She stated:

*I was better at influencing him. My mother and brother were not as good at this. I am his daughter, so I am better at this. When they encountered problems or had arguments with him…I would praise him and would also scold him. Hehehe. I adopted the carrot-and-stick approach because he was like a child.* (C1)

Another participant, a former professional caregiver, quit her job to care for her husband full-time. She stated:

*I took care of him [the patient] like a baby. I treated him as if he were a glass doll…an infant, [as though he was] a mother who had just given birth and was doing her recuperative month.* (C16)

#### Adjusting to a new pace of life

Cancer disrupts the sense of order and pace in a family's life, and family member roles and responsibilities may change as a result. If family members of the affected individual maintain favorable communications and coordination, this threatening and stressful crisis may offer an opportunity for them to commence new lives and enhance their relationships. Eight (approximately 40%) of the participants reported adjusting their pace of life by taking turns to take care of their patient and rearranged their daily schedule. A 65-year-old female participant who had taken care of her mother with lymphoma for 6 months reported striking a tacit agreement with her brothers:

*My younger brother visits my mother on Saturday, and my older brother visits on Sunday…. If I have to go out and do stuff, I can do so on Saturday, Saturday noon, or Sunday evening. I have two time slots when I can go out and buy stuff.* (C15)

Another participant stated she and her brother took turns caring for their father with esophageal cancer for 2 months. Taking turns to care for him and sharing their responsibilities gave them opportunities for respite. She stated:

*I feel that, so far (uplifting tone), for example, we must take turns caring [for the patient]. I feel that having sufficient rest is necessary for us to have the strength to provide better care. Therefore, I think that we must support each other by doing our part. That's why we implemented shifts.* (C5)

Similarly, a woman with colorectal cancer who had been receiving treatment for approximately 1 year was cared for by her youngest daughter, who was her primary caregiver. The daughter's siblings took turns caring for their mother during the holidays. The participant stated that the best change in her life was the additional time she had to visit her mother and that the relationships between her family members had improved. She stated:

*…Life has changed. Specifically, in the past, we occasionally visited our mother to see how she was doing. Now, things have changed…. I visit her every day, and I also visit her on Saturdays and Sundays…. Every week, I play mahjong with her, and she is happy.* (C6)

## Discussion

The focus of this qualitative study was on the care experiences of FCs caring for their older family members with cancer. All of the cancer patients were undergoing active cancer therapy in a chemotherapy outpatient setting. Twenty FCs were recruited and enrolled as participants in this study. They reported a need for nutrition-related guidelines and correct medical knowledge to better care for their patients at home. The finding regarding nutrition echoes a previous study that found nutrition to be a major concern of older patients with cancer because of physical function decline, comorbidities, and treatment-related side effects such as vomiting, mucositis, and nausea (Blasiak et al., 2020). Older patients with cancer who are malnourished face an up to 1.87 times higher risk of all-cause mortality (Zhang et al., 2021). In this study, all of the older patients with cancer received chemotherapy only or chemotherapy combined with other cancer therapies such as radiotherapy, targeted therapy, and immunotherapy. Twelve (60%) of the patients were affected by common chronic diseases, including hypertension, diabetes mellitus, and heart disease. This led the participants to highlight the need for professional healthcare teams to consider the side effects of cancer therapies and the concomitant effects of chronic diseases to create a multidisciplinary approach for menu preparation that includes variety and easily chewable foods rich in protein. In a prior study in a Western setting, the challenges of preparing food and meals at home were noted from the patients' perspective (Hestevik et al., 2020). However, because of the expectations surrounding caring for sick family members in the Chinese cultural context (Ho, 1994), FCs are the primary individuals responsible for preparing the food served to sick family members. Therefore, the participants in this study rather than the patients struggled with how to prepare diverse meals. FCs should be supported or provided with nutrition education tailored to their cultural expectations and preferences.

With regard to medical knowledge, as in Sherman et al. (2014), this study found lack of professional medical information and support to be an intense source of distress. The participants described feeling overwhelmed in the face of unexpected information and needing more time and medical information to understand their family member's cancer diagnosis and to determine which therapy was the better choice. As treatments progress and disease status changes, the provision of correct information necessary to make informed treatment decisions becomes a major concern for caregivers (Sherman et al., 2014). In addition, clinicians should share the best available evidence on treatment options to help patients make appropriate decisions (Elwyn et al., 2010). On the basis of the findings of this study, medical knowledge and evidence should be regularly provided to FCs to assist older family members, particularly older patients with cancer, to make treatment decisions together.

In addition, this study found that the participants perceived emotional tension while providing care related to such issues as being unable to communicate with a patient with poor emotional regulation, worrying about whether the patient might die suddenly, and losing patience (particularly in participants who were the spouse of the care recipient). This finding supports previous studies that found FCs who were the spouse of their care recipient to face a 14 times higher risk of depression than nonspouse relatives (Lee et al., 2013) and quality of life in FCs to be influenced by the mood of their care recipient (Shahi et al., 2014). Also in this study, similar to Schulz et al. (2020), we found that older FCs caring for older patients with cancer at home face huge challenges that should be addressed by appropriate intervention strategies. Moreover, previous studies have reported that prolonged caregiving may impact the behavioral health of caregivers, causing higher levels of psychological distress (Grant et al., 2013; Y. Kim et al., 2012; Ochoa et al., 2020). The tense relationship previously shown to exist between patients and caregivers in terms of emotions and communications (Mosher et al., 2016) is also supported by this study. Participants who had provided care for longer periods tended to share that they more frequently expressed impatience and frequently felt tired because of their care recipients and their own emotional stress. Thus, providing social support, particularly emotional support and practical support such as respite care service, to FCs is important.

In this study, we also found that most caregivers found methods to relieve stress and rebalance their life. These methods included finding ways to relax by themselves, praying to feel calm, changing the way they interacted with the patient, and adjusting their pace of life. Some of these findings are consistent with previous studies (Sherman et al., 2014; Teskereci & Kulakaç, 2018) that found FCs successfully adopted coping strategies such as engaging in laughter, focusing on the positive aspects of their lives (Sherman et al., 2014), seeking spiritual support, praying to find inner peace (Teskereci & Kulakaç, 2018), and having someone to share care responsibilities. FCs communicate with their other family members for ideas, experiences, and support (Sherman et al., 2014), and these experiences may improve their ability to cope with their care recipient's illnesses (Tiete et al., 2021) and help them adjust their own life. Moreover, this study revealed interesting findings regarding how the participants adjusted how they interacted with their care recipient. Older patients were cared for like a baby by some of the participants. This may be a good strategy for communicating with patients, particularly those who are older.

### Limitations and Recommendations

The home-care experience of FCs in this study may differ significantly from the experience of caregivers who provide care during hospitalization. In addition, the results may not be generalizable to caregivers in other countries, as this study was focused on northern Taiwan. Educational interventions should focus on finding appropriate ways for FCs to interact with their patients and effective approaches to relieving stress in FCs and readjusting the rhythm of their family life.

### Conclusions

The caregivers in this study expressed a need for nutrition and medical-professional-knowledge-related guidance to better care for older patients with cancer at home. In addition, the results highlight a need to better educate these caregivers on how to regulate their stress and emotions and implement effective coping strategies, all of which are important during long-term care. Healthcare staff should remind FCs of the importance of continuing care after family members return home. This may be facilitated by healthcare staff understanding the actual experiences of families, both generally and in-depth, and developing discharge plans and family support networks that meet the needs of FCs and alleviate their burdens when caring for older patients with cancer.
